# Flexible competency framework: A tool for optimizing life science training

**DOI:** 10.1371/journal.pbio.3003331

**Published:** 2025-08-27

**Authors:** Xiuqi Li, Jelena Patrnogić, David Van Vactor

**Affiliations:** Department of Cell Biology, Harvard Medical School, Boston, Massachusetts, United States of America

## Abstract

Life sciences research requires high standards of rigor and professionalism, which need to be woven into the training future scientists. This Community Page presents a competency-based framework to use to align curriculum and trainee outcomes with educational objectives in the life sciences.

## Teaser

As life sciences research evolves, standards of rigor and professionalism remain essential. We present a flexible competency-based framework designed to align program objectives, curricula, and mentoring expectations, thereby enhancing trainee skill development and program adaptability.

## Introduction

The 21st century has been a transformative era in biomedical research, offering unparalleled opportunities to improve human health and well-being. Realizing this potential requires a robust and sustained science, technology, engineering, and medicine (STEM) education pipeline that prepares future scientific leaders. Although professional training in the life sciences continues to be anchored in the apprenticeship model, it has evolved significantly with the expansion of postdoctoral training and the call for training standards in research rigor, reproducibility, and responsibility, as championed by the US National Institutes of Health and other organizations [1, 2]. Defining and implementing these standards remains a key challenge.

In contrast to fields such as law and medicine, which have long relied on credentialing systems (*e.g.*, bar or board exams) based on a consensus inventory of operational knowledge and capability, biomedical science has been slow to adopt universal standards of competency. Recent efforts to modernize and innovate biomedical training have focused on enhancing mentorship [3], refining training objectives [4], and improving career readiness [5, 6]. Moreover, tools like individual development plans (IDPs), initially adapted from the business sector for use with STEM postdoctoral trainees [7, 8], are increasingly being used to track scientific and professional development across STEM career stages. In this context, we describe our current efforts to harness a competency framework to better align training goals and outcomes and support STEM trainees across their career development.

## A competency framework for training in biological and biomedical sciences

Well-defined knowledge and competency inventories have long been used to structure curricula and benchmark assessments from school to university education [9, 10]. However, as students transition to graduate and postdoctoral training—where they tackle more open-ended research questions with increasing independence—formal structure often diminishes. In a recent process of self-study at our institution focused on rigor and transparency [11], many colleagues reached a consensus that an explicit research competency framework could improve the design and evaluation of advanced-stage scientific training. This prompted us to pilot the use of such a framework in our largest PhD training program, Biological and Biomedical Sciences (BBS).

**Fig 1:**
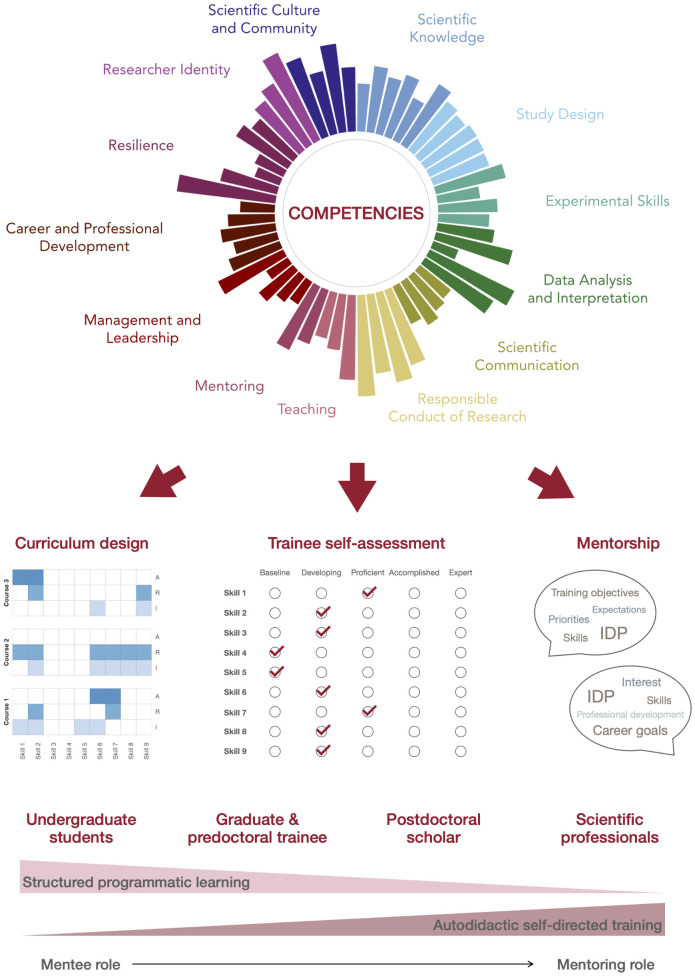
Using a competency framework to align curriculum, self-assessment, and mentorship in life science training. Conceptual diagram illustrating a competency framework comprising 56 competencies across 13 core areas (top, see our preprint [12] for more details). This framework can serve as a foundation for curriculum mapping, trainee self-reflection, and a shared language for mentoring (middle). We are currently piloting our competency framework as an iterative self-reflection process for trainees, using a structured self-assessment survey. Trainees rate themselves across competencies, identify areas for growth, and receive confidential reports of their responses to inform goal-setting discussions with mentors. This approach aims to encourage tailored skill development, ongoing dialogue, and documentation of progress from matriculation to graduation. In the future, we could imagine including parallel mentor evaluation as part of a formative assessment. Applying this approach across training stages could promote intentional, integrated trainee development as they transition from structured coursework to self-directed research and professional growth (bottom).

The new competency framework for BBS, described in a recent preprint [12], draws from national guidance, including recommendations from the Association of American Medical Colleges [4] and others [1, 2], but was adapted to reflect our program’s specific training goals. It outlines 56 competencies grouped into 13 core areas, ranging from ‘Scientific Knowledge’ and ‘Study Design’ to essential professional skills, including ‘Communication’, ‘Leadership’, and ‘Resilience’ (Fig 1). Unlike traditional, knowledge-centric curricula, this approach emphasizes the importance of a broad, rigorous, and transferable skill set to support the holistic development of trainees, with particular consideration of the expanding range of career paths pursued by biomedical PhD graduates [6, 13, 14].

The shift toward a skills-based framework reflects a broader recognition across the scientific training community that preparing trainees for today’s broad range of career opportunities requires more than deep subject matter expertise. PhD graduates now pursue rapidly evolving roles across industry, nonprofit organizations, and education sectors [13, 14], so our training programs need to incorporate competencies that extend beyond the laboratory. In this way, a competency framework can offer a flexible yet structured way to guide curriculum design, promote alignment across training stages, and support the various needs of trainees (Box 1). Indeed, we have applied the framework to map and critically evaluate our own curriculum [12] and to compare our programmatic objectives to student self-efficacy using the same rubric of skills (Fig 1). By using the same competency framework to guide both course design and individualized assessment with a customized IDP, we aim to support PhD students as they transition from structured coursework to independent research. Additionally, because IDPs are also used for annual benchmarking of postdoctoral training [7, 8], we have proposed extending this competency-based self-assessment to postdoctoral fellows at our institution. Embedding this approach across training stages can guide more nuanced and personalized evaluation of skill development, supporting informed mentoring, tailored goal setting, and the self-directed learning essential for careers as independent scientists.

**Box 1. Application of the competency framework**

The hypothetical cases below demonstrate how the competency framework can facilitate trainee self-assessment, structured conversations with their mentors, and joint goal setting for skills development. These cases draw upon our own and our colleagues’ mentoring experiences as well as scenarios often encountered in pre-doctoral and postdoctoral training.

**Case 1**

A postdoctoral fellow, who is interested in pursuing a career in regulatory science, recognizes that their ‘Scientific Communication’ skillset needs to extend beyond academic publishing to regulatory document writing. Their primary research mentor excels at academic writing but lacks expertise in this specialized area. During their annual IDP meeting with their mentor, the postdoctoral fellow identifies this gap and asks the mentor to facilitate a connection with another mentor in the university’s technology transfer office. This enables the postdoctoral fellow to gain practical experience and receive targeted feedback on regulatory writing, thereby achieving this specialized skillset through a mentoring network approach.

**Case 2**

A graduate student, while strong in laboratory techniques, realizes through self-assessment that they lack experience with ‘Project Management’, a gap their principal investigator (PI) does not address, because the PI’s mentorship primarily focuses on experimental design. Recognizing this need, the student enrolls in university-offered workshops on project management software that sparks their interest in potential industry careers. The student goes further and seeks informational interviews about project manager roles in industry. The PI and the student’s thesis advisory committee, guided by the holistic competency framework integrated into the annual IDP, support these efforts, acknowledging their importance for the student’s career goals after discussing the student’s career aspirations.

**Case 3**

A graduate student working on a project involving high-throughput sequencing data realizes that their ‘Data Analysis and Interpretation’ skillset should include proficiency in bioinformatics, computational tools, and other data science skills. Their primary research mentor, while experienced in traditional molecular biology techniques, lacks expertise in computational analysis. During regular check-ins, the student and their mentor jointly decide that the student should dedicate some time to developing these skills. With guidance from their mentor, the student connects with the university’s bioinformatics core facility and enrolls in a specialized summer course on high-performance computing at a different institution. These actions enable the student to develop the specific analytical skills needed, enhance their research, and build a foundation for future projects involving complex datasets, while making valuable connections with computational biologists and identifying a potential postdoctoral advisor.

## Cultivating rigor and transparency through laboratory culture and mentorship

For most biomedical trainees, the core of their research training remains rooted in the apprenticeship model of laboratory research. As trainees transition from structured curricula to open-ended research inquiry, they learn to become self-directed scholars (Fig 1). Their continued development is shaped profoundly through daily interactions with mentors, peers, and the scientific process itself, underscoring the importance of quality mentorship and positive laboratory culture. Yet PIs—central figures in shaping this environment—bring varied backgrounds and levels of mentoring experience. While formal standards in mentoring practice have helped define best practices [3], mentors may still need additional resources and guidance to cultivate an inclusive, intentional, and growth-oriented environment.

A shared competency framework can serve as a valuable tool in this context. By making developmental expectations explicit, the competency framework can support mentors and trainees in articulating goals, tracking progress, and staying focused on the full arc of scientific and professional development. For PIs, it can complement existing supervisory responsibilities by providing structure for regular check-ins, formative feedback, joint goal setting, and the validation and documentation of skill development. Rather than relying on informal norms, mentors can use the framework to align day-to-day lab activities with broader training goals. At the same time, the framework can help illuminate the value of mentoring networks, enabling trainees to engage with multiple mentors who contribute complementary expertise, perspectives, and career insights (Box 1). In many labs, postdoctoral researchers play a critical role in this network, serving as both near-peer mentors and developing scientists who benefit from the same intentional support. As recommended by colleagues during the self-study process at our institution [11], embedding competency development into the fabric of lab life could help strengthen mentorship and reinforce scientific values such as rigor, reproducibility, and transparency. In doing so, these values move from abstract principles introduced in classroom settings to lived practices enacted within the laboratory environment.

## Aligning the training mission with the national landscape

As the job market evolves, training programs that remain attuned to the dynamic career trajectories of today’s biomedical PhD graduates are likely to attract capable and ambitious candidates. The modular design of our competency framework [12], with core areas and a nested hierarchical structure within each, makes it readily adaptable. Distinct programs can tailor and expand their individualized competency frameworks to reflect the practical demands of the most productive career paths, including different roles in industry and academic research. For example, competencies related to regulatory or data science, project management, and public engagement may be especially relevant to trainees pursuing careers outside of academic roles. Regular dialogue with employers could guide such refinements, helping ensure that the competency framework remains aligned with workforce expectations.

A separate but related consideration is how programmatic training objectives respond to evolving national priorities and standards in scientific research. Training programs vary widely in their format, structure, and requirements—not only across institutions in the US, but also internationally—reflecting different academic traditions, funding structures, and workforce needs. While regional accreditation bodies provide broad criteria for assessing graduate degree-granting programs, these standards typically stop short of requiring specific inventories of professional competencies. The complexity of degree programs makes this impractical. Our competency framework could offer a flexible, unifying meta-structure and a shared language for intentional programmatic design. It is possible that incorporating such a competency framework into the self-study process during accreditation reviews could enhance the transparency and evaluability of graduate training, allowing reviewers and other stakeholders to better understand how individual program elements are purposefully organized to support trainees for productive entry into the professional sphere.

## Conclusions

As the landscape of biomedical research continues to evolve, so too must the systems that prepare the next generation of scientists. The use of a competency framework across curriculum design, trainee self-assessment, and mentorship represents one step towards more integrated and intentional training in the biomedical sciences. While no single model can capture the complexity of individual learning paths, a shared structure can offer valuable guidance for trainees navigating their development, for mentors supporting that growth, and for programs striving to adapt to a rapidly changing research landscape.

## Abbreviations

BBS: Biological and Biomedical Sciences
IDPs: individual development plans
PI: principal investigator
STEM: science, technology, engineering, and medicine
